# Schistosomiasis in African infants and preschool children: let them now be treated!

**DOI:** 10.1016/j.pt.2013.02.001

**Published:** 2013-04

**Authors:** J. Russell Stothard, José C. Sousa-Figueiredo, Martha Betson, Amaya Bustinduy, Jutta Reinhard-Rupp

**Affiliations:** 1 Parasitology Department, Liverpool School of Tropical Medicine, Pembroke Place, Liverpool, L3 5QA, UK; 2 Department of Infectious and Tropical Diseases, London School of Hygiene and Tropical Medicine, Keppel Street, London WC1E 7HT, UK; 3 Department of Production and Population Health, The Royal Veterinary College, Hatfield, Hertfordshire, AL9 7TA, UK; 4 Department of External Innovation, Global Research and Early Development, Merck Serono S.A. Chemin des Mines 9, CH-1202 Geneva, Switzerland

**Keywords:** neglected tropical diseases, preventive chemotherapy, paediatrics, praziquantel, cure rates, pharmacokinetics, malaria, HIV

## Abstract

The occurrence of schistosomiasis within African infants and preschool children has been much better documented in recent years, revealing an important burden of disease previously overlooked. Despite mounting evidence showing that treatment with praziquantel is safe, beneficial, and could be delivered within ongoing public health interventions, young children still do not have satisfactory access to this drug, and a significant treatment gap exists. Progress towards resolution of this unfortunate health inequity is highlighted, including the development of an appropriate paediatric praziquantel formulation, and present blocks are identified on securing this issue within the international health agenda.

## Mind the gap: the need for praziquantel

Five years ago, the treatment needs of African infants and preschool children afflicted by schistosomiasis (see Glossary) with the anthelminthic praziquantel (PZQ) were raised indicating that national control programmes (NCPs) should consider more formally the health status of these younger children [1–3]. It was then an open question as to whether these younger children could or should be included within preventive chemotherapy (PC) campaigns. Since then there have been significant developments to better document this disease burden by the retargeting of epidemiological surveys and to better tailor international policies and practices towards this paediatric setting [4–10].

As several studies come to fruition and as new evidence emerges, a fresh international dialogue has been established with key support and coordination offered by the World Health Organisation (WHO). This is to ensure the development of a longer-term framework to manage this disease at the individual and (or) community level. In so doing, these studies have heightened the realisation that control of schistosomiasis in young children has some unique challenges [7–10]. These include needs for better diagnostics, revised licensing of PZQ, scaled-up deployment of available and future PZQ formulation(s), and sustaining associated drug delivery systems for integrated control of neglected tropical diseases (NTDs) [5,9].

**Figure 1:**
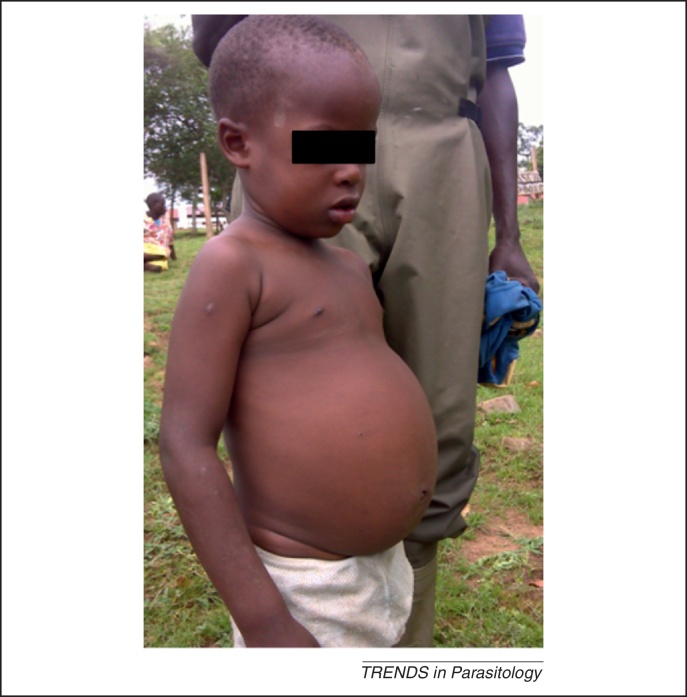
Advanced intestinal schistosomiasis in an 8-year-old child. This young child was encountered during general disease surveillance activities on the Sesse Islands, Lake Victoria in November 2010. Clinical presentations such as this are still common throughout endemic areas of Uganda [60] and warrant better formal recording if the burden of morbidity in young children is to be quantified. Sadly, although this child may have received his first praziquantel (PZQ) treatment within primary school, his individual morbidity is already at an advanced stage and may not be fully reversible. Had this child had treatment at a preschool age this might have been averted, which is perhaps a strong indictment that further inaction is unethical.

A key driver behind this new impetus is that overt and cryptic morbidity commonly attributable to schistosomiasis can occur during school age (Figure 1). Any delay in prompt access to treatment of these younger children points towards an unfavourable downstream clinical outcome. Even more importantly is that at the individual level, these infections cause detrimental onslaughts to the health of the child during key developmental stages. Moreover, as NCPs strive towards elimination of infections by PC alone, or in combination with other measures at the community level, promotion of access to PZQ treatment for any infected case, irrespective of their age, becomes ever more important [11–13]. Conceptually, this is to ensure that general reductions in parasite transmission are consolidated across all community groups [14,15]. Thus, the need for equitable treatment of younger children has become so obvious that a ‘PZQ treatment gap’ terminology has been coined [16]. Although mentioned in the WHO 2012–2020 strategic plan, there are still significant blocks to overcome to embed this issue firmly within the agenda of NTD control and to see National Control Programmes embrace this element [17].

## First blocks off on PZQ treatment: application and dosing

Although PZQ is perhaps one of the safest drugs being used in PC campaigns, there was little formal legislation and documentation confirming its use in younger children [18–20]. An age-related cut-off in children aged below 4 years existed simply due, perhaps, to an incomplete registration and limited future vision of treatment needs by Bayer and Merck (see below). Thus, PZQ is administered in ‘off-label’ settings, which make international agencies rather uncomfortable without substantial evidence to the contrary, and with legal indemnity falling upon those who have chosen to use it under their own discretion. Although this ‘off-label’ phenomenon is often common practice in paediatric ward settings [21], it did little to assuage the initial ethical, legal, and insurance-driven arguments against its extended use; a parallel predicament was the use of PZQ in pregnancy, which is now endorsed by the WHO in their revised guidelines [17].

These counter points of view were much diminished, after a tipping point was reached when the burden of disease in young children was firmly demonstrated as being so alarming that any further inaction should be considered unethical. There are four key medical tenets appropriate here: beneficence, non-maleficence, autonomy, and justice [22], all of which have some bearing upon how we react to this PZQ treatment gap. Nonetheless how much more convincing evidence was needed? With the help of the WHO, a cross-country study was undertaken in Mali, Niger, Sudan, Uganda, Zimbabwe, and Egypt in 2009 [4,7,23,24]. Here it was the intention to take more formal steps to develop a portfolio of evidence reporting on its use in terms of drug safety and performance, although using crushed or broken tablets versus a liquid suspension. This was not a formal clinical trial, which has a totally different set of legal, financial, and registration requirements, but rather an on-the-ground exploration with the aim of remedying a glaring health inequality. This was a particularly seminal and brave first step by the WHO and was warmly welcomed by the in-country coordinators seeding the need for further applied research.

Many of the areas where schistosomiasis in preschool children abounds are often characterised by impoverished or under-resourced local static health systems [25,26]. For example, despite trained staff, in rural health clinics there is often no provision of accurate weighing scales or the availability of fully functioning diagnostic facilities (i.e., urine and faecal examination kits and access to compound microscopy). Resolution of this weight-based bottleneck was achieved in general PC campaigns through the use of a height pole or treatment stick. Until 2012, the existing height pole was inadequate for dosing children under 94 cm in stature. Following the work of Sousa-Figueiredo *et al.* [9], this has now been comprehensively resolved with an extensive validation of a downwardly extended height pole offering dosing at 40 mg/kg at 0.5 (½), 0.75 (¾) and single tablet divisions (Box 1). This of course needs tablets which are scored with quarter divisions, that is, breakable sections each of 150 mg, which are not always fabricated in this manner by all tablet producers. It is open to conjecture whether this 40 mg/kg dosing, which is a direct extrapolation from adults, is appropriate for young children as generally a nonlinear relationships exists. Validation of this simple dosing regimen awaits the collection of future pharmacokinetic and pharmacodynamic evidence which may encourage the use of 60 mg/kg (see below) or even higher levels, and further consideration in a revised drug licensing schedule.

## Second blocks off on PZQ treatment: taste and formulation

Other concerns about administration of PZQ include the difficulties of children swallowing the large, cumbersome and bitter-tasting tablets, where risk of rejection and possibilities of choking should be considered [6,24]. Tablets can be easily crushed and mixed with honey or flavoured juice to make them more palatable and easily swallowed [26]. Of note is that the unusual flavour of PZQ (a racemate of *dextro*- and *laevo*-isomers) is not always recognised by younger children as it is in older counterparts or adults, perhaps due to a maturing palate of the developing child or inexperience of flavours. A liquid suspension of PZQ (Epiquantel^®^) is manufactured by EIPICO and licensed in Egypt for school children. Epiquantel^®^ is frequently referred to as a syrup formulation, for its flavour is strongly masked by aniseed. This in itself is not to the taste of all. Contraindications include use in patients with ocular cysticercosis or those with a general intolerance to PZQ and breast-feeding mothers should suspend giving their breast milk to children for 72 h after treatment [4].

The Epiquantel^®^ formulation comes in 15 ml dark glass bottles with a concentration of 600 mg of PZQ per 5 ml of fully mixed suspension. For dosing, each bottle also comes with a small plastic measuring cup with a 5 ml division, conveniently capping the sealed metal bottle top. As part of the six-country WHO study, although there were some positive considerations to its use, in terms of acceptability and parasitological performance it was judged to be no better than crushed or broken tablet alternatives [4]. Disadvantages included the large packing and storage volume which was cumbersome in comparison with existing tablets. This caused significant problems in logistics even in the small-scale study, predicting later problems in large-scale supply chain management and distribution. Perhaps more importantly, however, was the limited availability of Epiquantel^®^, which is produced in an *ad hoc* manner by EIPICO and is an expensive alternative making it financially unattractive. In line with new WHO general guidelines for paediatric medicines that promote the use of water-dispersible tablets, it was concluded that Epiquantel^®^ offered no significant improvement over crushed or broken tablets and that the latter should be continued to be used until an appropriate paediatric formulation becomes available [4]. Another recommendation was that PZQ could be delivered and administered to afflicted communities on the back of the Expanded Programme of Immunisation (EPI) campaigns which have highly proven levels of outreach into sub-Saharan African communities as supported by major international agencies (e.g., UNICEF) [4].

## Focus on Uganda: evidence from the SIMI cohort

November 2012 saw the end of a 4-year project funded by the Wellcome Trust entitled Schistosomiasis in Mothers and Infants (SIMI) [26]. The major aim of SIMI was to conduct a novel longitudinal cohort study assessing the need for and performance of crushed PZQ in terms of safety and parasitological cure in six villages on the lakeshore of Albert and Victoria. The strategy behind this study was to emulate future PC within this setting but also to assess how often young children should be retreated. It may be, for example, that a single PZQ treatment might be sufficient to stamp out their infections over a 2-year period. Retreatment periods need to be set against actual levels of reinfection which in turn need to be assessed with presently available diagnostics [16].

Over an 18-month period, the children (*n* = 1211) and their mothers were followed at five time points. Key results show that nearly half of the children aged 3 months and above have active infections (Table 1) [16]. More startling was that the levels of reinfection, especially on the Lake Albert shoreline, were so substantial such that even within a 6-month period following from two PZQ administrations had little concrete impact in terms of diminishing local prevalence. The situation in Lake Victoria was more favourable, probably due to a slower general transmission dynamic [16]. Assessing the risk of infection and levels of reinfection in young children is problematic owing to the distinctions between active and passive water contact mechanisms [1]. A pilot study in Lake Albert attempted to explain why transmission seemed to be so high. Using a novel application of global position system (GPS) data loggers (Box 2), young children were shown to typically have up to 30 min of active daily water contact in addition to more cryptic passive water contact levels [27]. When taken together, however, this revealed an astonishing amount of water contact risk and pointed towards a stable but previously cryptic transmission group within the community.

In search of the best diagnostics, a combination approach was taken bringing to the field best available tools [16,28–30]. For faecal examination, consecutive 2-day stool specimens were collected and each examined by duplicate Kato–Katz thick smears with microscopy. To bolster this, urine circulating cathodic antigen (CCA) dipsticks were used together with analysis of sera from clotted finger-prick blood using a commercially available ELISA kit, detecting host IgM/G antibodies to schistosome soluble egg antigen (SEA). Initial studies within a subset of children, when additional faecal concentration examination techniques were applied, found that schistosome antigens in urine, eggs in stool, and host antibodies to eggs demonstrated a general prevalence of 47.5%, by a positive criterion from at least one diagnostic method. Although children as young as 6 months old could be found infected, the average age of infected children was between 3.25 and 3.75 years, when diagnostic techniques all became broadly congruent [31]. The prevalence of intestinal schistosomiasis across the five time points is shown in Table 1, which despite treatment with PZQ shows little tangible signs of abating and without treatment would have been more alarming [16].

Another open question was selection of which diagnostic tool to choose to best track the reinfection dynamics across these children [32]. This might also prove useful for a future selective treatment rather than a mass treatment approach. Diagnostic scores from a total of 925 children across a 12-month period were available for comparison. As a point-of-care diagnosis, the urine CCA dipstick test achieved sensitivity and specificity values ranging from 52.5–63.2% to 57.7–75.6%, respectively, with faecal microscopy achieving very high specificities (>87%) but sensitivities as low as 16.7% upon comparison to pooled diagnostic scores per child. The urine CCA test was shown to be more effective than faecal microscopy of a single stool sample and was not significantly impacted by PZQ treatment history or coinfections with other intestinal helminths [32]. Although its outlook in application is promising, it is yet to be proven how the urine CCA test could be best used in future mapping or selective treatment initiatives given a general reluctance of many implementation agencies failing to embrace new and appropriate technologies as they appear [20,33,34].

## Consequences of early schistosomiasis: old markers of morbidity

Faecal or urine egg counts have been the cornerstone of measurements associated with morbidity in school children for many years [33]. Whether they are entirely appropriate in preschool children is open to conjecture. More broadly, there is a limited repertoire of field-applicable tests for direct assessment of morbidity [35] and little application of these in the setting of young children where infections are typically of ‘light intensity’ at least on the basis of captured egg excretion using standard urine or faecal sampling. Nonetheless, macrohaematuria, microhaematuria, and proteinuria (as assessed by reagent strips), and other urine abnormalities, have been found in young children in *Schistosoma haematobium*-endemic communities [7,6,36–38]. Perhaps the most surprising was the study by Sacko *et al.* who documented astonishing levels of upper and lower urinary tract damage as seen upon ultrasonography, some of which was still persistent after PZQ treatment [4].

In *Schistosoma mansoni*-endemic areas, visible or reported blood in stool (as described by the child's mother) has been documented [39,40]. Liver and spleen damage at this early age is known by palpation of enlarged and texture-hardened organs [41,42]. Liver fibrosis and periportal thickening has been observed in infants and preschoolers in *S. mansoni*-infected communities [41,43]. Although anaemia is often associated with schistosomiasis, assessing this in younger children is confounded by coinfections, most notably malaria. Recent work in young Ugandan children has found that anaemia is mostly attributable to malaria rather than intestinal schistosomiasis [44]. By contrast, in West Africa, there was some evidence of an association between anaemia and *S. haematobium* infection in younger children [45].

## Consequences of early schistosomiasis: new markers of morbidity

In a novel attempt to identify markers of intestinal morbidity in young children, Betson *et al.* field-tested faecal calprotectin ELISAs and faecal occult blood (FOB) rapid diagnostic tests (RDTs) concurrently with questionnaire-based methods enquiring about a history of blood in stool, diarrhoea, and abdominal pain (as reported by the child's mother). Although no association between egg-patent schistosomiasis and faecal calprotectin was observed, a strong positive association with FOB and symptoms reported by mothers was detected [40]. The association between *S. mansoni* infection and FOB (but not symptoms reported by the mothers) was maintained after one or two doses of PZQ over a 12-month study period [39]. This suggested that the FOB RDT was a useful marker for assessing intestinal morbidity in young children at the community level and monitoring changes in morbidity after mass PZQ treatment. Although the production and availability of FOB RDTs is insecure, even within the UK, they have again been used in Uganda during a PZQ efficacy study where declines in FOB were observed 24 days after treatment (A. Bustinduy *et al.*, unpublished). The field technology for detection of faecal calprotectin has also changed with the availability of a commercially available RDT and cassette reader which has overcome several cumbersome aspects of ELISA. With some exceptions, faecal calprotectin levels seem to increase 24 days after PZQ treatment pointing towards rather complicated inflammation biology of the mucosal surface of the bowel (A. Bustinduy *et al.*, unpublished).

A novel field-applicable approach for assessing urinary tract and bladder wall pathologies in *S. haematobium*-endemic communities is to measure urine albumin levels using an Albumin-HemoCue photometer [46]. Raised albuminuria was shown to be a promising marker of pathology in school children and more recently albuminuria was found to be associated with both egg-patent *S. haematobium* infection and microhaematuria in young children in Malawi (H. Poole, MSc thesis, Liverpool School of Tropical Medicine, 2012). In this setting, albuminuria was also strongly correlated with infected cases as identified by serology, suggestive almost of equal diagnostic sensitivity and specificity. This latter finding is particularly exciting as the search for the most sensitive point-of-care test for urogenital schistosomiasis continues, which will also probably have bearing on detection methods for female genital schistosomiasis [47].

## Early schistosomiasis: chronic infections and poor cure rates

Evidence from the WHO six cross-country study showed that cure rates with PZQ were satisfactory [4] but intestinal schistosomiasis is a persistent and chronic infection in young children in the lakeshore settings of Uganda (Table 1) [16]. It is therefore perhaps ambitious to assume that PZQ alone will be able to eliminate sufficiently all infections in this setting without recourse to more aggressive use of treatments and complimentary measures [48]. It is known, for example, that the present administration and dosing at 40 mg/kg does not always effect a full parasitological cure. This is often alongside coadministration of artemisinin-based combination therapy (ACT) for management of concurrent malaria, where the artemether component is thought to have some antischistosome activity on juvenile worms [49], but this appears unfounded at the current antimalarial dosing in the SIMI cohort [16].

From a total of 369 children found to be egg-patent for intestinal schistosomiasis, 305 were followed up 3 to 4 weeks after PZQ treatment and infection status reassessed using a variety of methods. Although the overall observed parasitological cure was 56.4%, a significant difference was found between a subset of children who had a history of multiple PZQ treatments (between one and four administrations over an 18-month period), where cure rate was 41.7%, and those who had never received treatment (cure rate was 77.6%) [10]. The cure rate was clearly lower in younger children (<3 years of age) and in those with a history of previous treatment. Cure rate, but not egg reduction rate, was also lower in children with heavier preintervention infection intensity. Recent work in Ugandan children suggests that higher than recommended doses of PZQ may be warranted for *S. mansoni* treatment. The difference in egg-clearance cure rate 4 weeks after treatment was 82% compared with 68% for different dosing strategies (60 mg/kg vs 40 mg/kg). Egg reduction rates ranged from 91% to 82% in the 60 mg/kg and 40 mg/kg arms, respectively. Further pharmacokinetic data are soon to follow and may well challenge the current treatment recommendations for *S. mansoni* in children of all ages (A. Bustinduy *et al.*, unpublished).

Rather unsatisfactory cure rates in young children have now been reported in Niger. Using Epiquantel^®^, moderate to high levels of efficacy against *S. haematobium* were found with cure rates of 85.7%. This contrasted sharply, however, with that for *S. mansoni* which was 50.6% against clearance [7]*.* The marked differences in species-specific drug performance call for further research into optimising treatment for each schistosome species whilst remaining mindful of any revised drug licensing [7,10]. There is also an ongoing debate concerning the suitability of using parasitological cure in terms of treatment campaigns, owing to a variety of issues best described in papers by Montresor *et al.* [11,50].

## Towards better access and formulations of PZQ

Before the reductions in price of PZQ there was actually a slow uptake of this drug as treatment of choice, the reasons for which were analysed by Reich and Govindaraj [51], when PZQ first entered the market, aspects related to its access and pricing had not been sufficiently addressed creating a lasting detrimental legacy. Other factors also played contributory roles: the reluctance of donor agencies in engaging early on in purchasing initiatives, failure of non-governmental organisation (NGO)-based implementation agencies to sustain control activities in the long term or expand to the national level, the lack of decision-making autonomy of low-income countries to leverage or synergise with donor agencies and rather simply put, many endemic countries lacked the infrastructure to participate appropriately in international tendering [51].

With the advent of the Schistosomiasis Control Initiative [52] some of these issues were resolved [53]. Until very recently, the global supply chain of PZQ was restricted [54], largely due to the available commercial producers being reluctant to engage in scaling-up of production until better estimates of global drug consumption/needs were in place to create a sustainable ‘market’ [17]. With the London Declaration on Neglected Tropical Diseases (LDNTDs) this picture has changed dramatically. A cardinal step was taken by the WHO in setting out a clear strategy of the scale-up of PC and assistance to countries to assess their future PZQ needs until 2020 and beyond, but these estimates do not factor in the treatment needs of infants and preschool children in their calculations [33].

Responding to these fundamental changes before the LDNTDs, in 2007 Merck (Germany) started its PZQ donation programme to the WHO, committing to donate 200 million tablets of PZQ over the next 10 years with the company increasing its donation, as spurred on by the LDNTDs meeting, to delivering 250 million tablets per year in the medium term. This would correspond to a treatment of approximately 100 million school children each year. Moreover, realising that this formulation was not particularly paediatric friendly, because off-label use (use beyond the terms of the license) outside of the donation programme implied that its performance in terms of efficacy and safety had not been sufficiently studied to ensure that the current formulation of PZQ product is delivering its full therapeutic potential. Merck has committed therefore to addressing the treatment needs of infants and preschool children by developing an appropriate paediatric formulation.

From a pharmaceutical perspective, this is a significant task and to close this PZQ treatment gap, Merck started a public–private partnership (PPP) together with Astellas Pharma, the Swiss Tropical and Public Health Institute, and TI Pharma, a non-profit organisation. The overall objective of this partnership is to develop a PZQ formulation that is appropriate for the treatment of children covering the age range of 3 months to up to 6 years with formal product licensing after full development [47].

The project is currently in the preclinical phase for development of the appropriate formulation and will enter clinical development by 2014. Future research necessary to complete its registration is to investigate the optimum PZQ dosage to ensure an effective worm kill, concurrently with improvements in its ease of administration, for example, by community drug distributors. However, the success of such initiatives will depend not only on the commitment of the partners but also on the creation and implementation of a favourable ground for dialogue and interaction between afflicted populations, researchers, and clinicians as well as the public sector (e.g., health systems, control programmes, drug regulators, etc.). In the longer term, financial incentives and mechanisms need to be put in place within the pharmaceutical sector to ensure that access to the new formulation is sustainable after completion of the PPP. Some of these future issues in this paediatric setting will parallel the previous problems in general access to PZQ as discussed comprehensively by Reich [55].

## Schistosomiasis alongside other paediatric infections

In Africa, polyparasitism is perhaps the norm rather than the exception [56]. This milieu of parasites, other infectious diseases, as well as underlying nutrition states frankly questions the fundamental *raison d’être* of monospecific interventions, especially when rolled out into a disease-endemic community. Of the long list of infections, two perhaps rank higher than most – HIV and malaria – each being well known in the context of mortality and morbidity of young children. Children born to HIV-infected mothers are at risk of acquiring the disease and once infected have a poor clinical outcome if infections are left unchecked. Infants are particularly prone to malaria due to a naïve immune system unable to deal with invading parasites that can quickly wreak havoc. Nonetheless, both diseases are amenable to chemotherapy, HIV with highly active antiretroviral therapy (HAART) and malaria with ACT, which has become the front-line treatment for home-based management of malaria.

Should use of PZQ be considered more holistically within a disease-endemic community when other intervention schemes are ongoing? All should agree that health programmes strive towards healthy children no matter where their illnesses come from; however, ambition and reality sometimes become confused when put into practice on the ground. Pairing certain treatment combinations might lead to overburdening fragile health systems when distributors of medications are stretched in time and resources [57]. From a biological perspective, there may be unforeseen drug–drug interactions which alter their pharmacokinetic or pharmacodynamic profiles leading to diminished performance [20,58,59]. Evidence-based medicine in this setting is particularly weak as there are few clinical trials conducted in healthy adult volunteers in endemic disease settings and even fewer so in the paediatric setting. Children should be the priority target, as a historically vulnerable population frequently neglected behind their adult counterparts, for longitudinal cohort studies and closely monitored field–epidemiological studies to ensure treatments are rolled out as safely as possible.

## Towards young children free from schistosomiasis

What steps are now needed to be taken to resolve this ‘PZQ treatment gap’? With the changing WHO guidelines and future availability of a paediatric-friendly formulation from PPP, there is much optimism for African children to have better access to medication. To scale-up, PZQ delivery systems need to be securely embedded within a strong public health platform such as EPI. To achieve this, there needs to be strong communication between international donors for provision of funds and synergism with effective governments with clear political will and commitment to maintain a robust delivery of medicines. Although there are concerns that PZQ might not be fully effective in the treatment of schistosomiasis, this should not be a block to roll out of treatment in younger children but more of an indictment to maintain a closer monitoring and surveillance system to achieve healthier children. So to close as succinctly as possible, let them now be treated!

## Figures and Tables

**Figure I:**
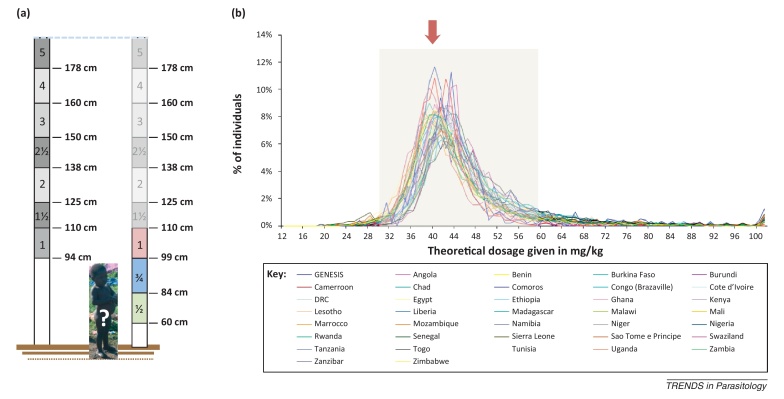
The current WHO height pole with a lower height threshold of 94 cm cannot be used for dosing children smaller than the one depicted and a downwardly extended dosing pole was needed for administration of PZQ for infants and preschool-aged children without recourse to weighing scales. **(a)** Original WHO (left) and extended (right) height pole with new 0.75 (¾) and 0.5 (½) tablet divisions. **(b)** Theoretical cross-country validation of the extended height pole with 99 cm, 84 cm, and 60 cm thresholds with best targeted dose of 40 mg/kg denoted by a red arrow, with acceptable dosing (30–60 mg/kg) indicated within the grey box.

**Figure I:**
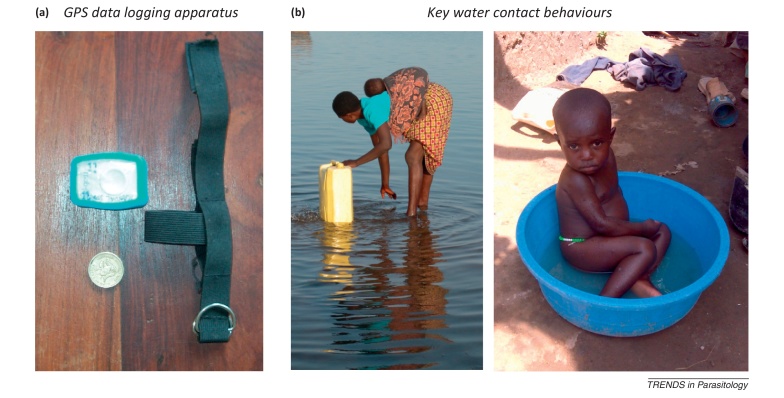
At an early age, first water contact typically occurs by passive mechanisms (i.e., being bathed) before a later gradual transition towards active mechanisms as the child is able to enter water alone. **(a)** The time, place, and duration of high-risk activities along the shoreline can be estimated by GPS data logging devices, which may be worn on the upper arm or wrist by study participants thus enabling a digital track-log of putative active water contact on the water margins. **(b)** GPS data logging approaches may, however, be confounded by specific behaviours, for example, (left) although the child may be in a high spatial risk zone, (s)he is not at immediate risk due to being carried; however, being bathed with jerry can collected water is a high-risk activity (right), although it is difficult to define spatially within the lake shoreline context.

**Table 1:** Prevalence of intestinal schistosomiasis across the SIMI young child cohort

Environment	Diagnostic	Baseline % (95% CI)	3-month % (95% CI)	6-month % (95% CI)	12-month % (95% CI)	18-month % (95% CI)
**Lake Albert**^a^	Kato–Katz	44.5% (40.3–48.7)	ND	30.7% (26.0–35.7)	37.6% (32.6–42.7)	Cohort closed
(*n* = 572)	Urine CCA	59.3% (54.9–63.5)	52.1% (47.4–56.7)	63.4% (58.4–68.2)	51.0% (45.9–56.2)	–
	SEA-ELISA	72.0% (68.1–75.7)	ND	83.2% (79.2–86.7)	87.2% (83.5–90.4)	–
**Lake Victoria**^a^	Kato–Katz	13.2% (10.8–16.0)	ND	7.5% (5.2–10.4)	14.5% (11.3–18.3)	11.1% (8.2–14.5)
(*n* = 639)	Urine CCA	43.2% (39.2–47.2)	39.7% (35.4–44.0)	40.2% (35.7–44.8)	40.7% (36.0–45.5)	34.2% (29.7–39.0)
	SEA-ELISA	40.8% (37.0–44.8)	ND	43.6% (39.2–48.2)	26.4% (22.3–30.8)	47.1% (42.3–51.9)

a In comparison to baseline, the average cohort recovery at each follow-up time point was 70%, that is, an initial loss of approximately 30% at 6-month follow-up thereupon with minimal subsequent drop-outs. During an 18-month study period, as estimated by different diagnostic methods, intestinal schistosomiasis is shown to be a chronic public health problem despite access to PZQ.

## Box 1 PZQ height pole: how low could you go?

In veterinary medicine, weighing facilities of large animals are typically lacking yet many anthelminthics are administered upon consideration of an animal's body weight. Thus, other biometric proxies of weight have been sought, height being the most important, which is often, but not always, strongly correlated to body weight. Hall *et al*. [61] first proposed the use of a height pole for administration of PZQ in children, as later endorsed by the WHO [62]. In its first design, the lowest height division was at 110 cm denoting 1.5 tablets and upon early experiences in African primary schools needed to be extended downwards to 94 cm offering a new height range for administration of 1 tablet to children in reception class. Further downward extension and biometric optimisation of this height pole was needed for even smaller children. Using biometric data from over 165 000 preschool-aged children (0–6 years of age, 60–110 cm in height) from 36 African countries, new tablet divisions were investigated to give dosing within the acceptable range of 30–60 mg/kg. This newly optimised dose pole (Figure Ia) offers dosing with a single 600 mg (height: 99–110 cm), 0.75 (¾) (height: 83–99 cm), and 0.5 (½) (height: 66–83 cm) tablet divisions, with over 95% of children receiving an acceptable dose (Figure Ib). In addition, by raising the single tablet threshold from 94 cm to 99 cm drug wastage could be reduced, which at the NCP level may have considerable drug savings [9,25,26]. These data also have bearings on the use of other height poles for treatment of young children with drugs that need consideration of body weight (e.g., antimalarials). Similarly, if doses of PZQ at differing levels are needed, that is, at 60 mg/kg, specific tailoring of an alternative height pole is possible. Nonetheless, care is needed when such tools are rolled out into field application to ensure that drug-specific height poles are not mixed up between applications.

## Box 2 Assessing risk of exposure in young children

For schistosome infection to take place requires the direct contact of skin and schistosome cercariae, notwithstanding the oral route of infection through the buccal mucosa. There has been a long history of assessment of environmental risk of schistosomiasis which has until recently relied upon observations of water contact as directly observed by field staff with tally sheets or on recalled histories of exposure upon interview questionnaire, both of which attempt to estimate skin immersion times. Young children present unique problems in being unable to answer questions and are generally less ambulatory in water than their older counterparts. The latter may have a subtle bearing on the efficacy of cercarial penetration as younger children stay relatively static in water margins being largely unable to swim and are simply content to dabble [1]. Estimating the level of exposure risk is important as it is often a predictor of reinfection and therefore the periodicity of retreatment with PZQ. By taking advantage of cheap GPS data logging technology and developing a custom-made Velcro arm strap (Figure Ia), Seto *et al.* [27] attempted to measure the levels of direct water contact of young children along the shoreline of Lake Albert, Uganda. Over a 3-day study period, time spent at the immediate lakeshore was calculated and found to be associated with egg-patent prevalence at baseline and subsequent parasitological follow-up. Comparing the GPS data logging patterns of mothers and children inferred that a social network of interpersonal interactions existed positively associated both with the number and duration of peer interaction, suggestive of strong sociocultural factors associated with water contact behaviour [63]. Although useful, this technology does have some limitations in imprecision as shown by the two photographs (Figure Ib). Quantifying the risk of exposure from directly drawn lake water in jerry cans is problematic but immersion studies using laboratory bred mice confirmed the occurrence of viable cercariae in the Lake Albert setting [64].

## Glossary

London Declaration on Neglected Tropical Diseases (LDNTDs): a high level advocacy meeting that took place in London in January 2012 that brought together international donors, the pharmaceutical sector, and implementation agencies to pledge support for development, donation, and distribution of drugs to combat NTDs. Further information can be found at http://unitingtocombatntds.org/downloads/press/ntd_event_london_declaration_on_ntds.pdf. A year later, the London Centre for Neglected Tropical Diseases was launched bringing together a new local partnership with Imperial College London (ICL), London School of Hygiene and Tropical Medicine (LSHTM), and The Natural History Museum (NHM) working within the UK Coalition against Neglected Tropical Diseases (http://ntd-coalition.blogspot.co.uk/).
Medical ethics: there are four central tenets to medical ethics. Beneficence (i.e., to do good), this will allow infected children, if treated, to achieve a better state of health given the alternative to absence of medication. Non-maleficence (i.e., do no harm), as PZQ has an excellent safety record, even in the absence of formalised randomised clinical trials, detrimental risks associated with treatment are negligible. Autonomy (i.e., self-determination), informing the mothers of infected children about the risks and benefits through informed consent addresses this principle. Justice (i.e., moral rightness), advocating the need for social responsibility to provide universal treatment care and treatment to those that need it.
Morbidity associated with schistosomiasis: disease is typically due to direct lesions by schistosome eggs and concurrent immunopathology to tissue granulomata. Manifestations for urogenital schistosomiasis include (macro)haematuria and (micro)haematuria where there is visual blood in the urine or cryptic amounts as detected by reagent strips, respectively. Urine reagent strips also detect proteinuria, the main biological component being albumin, which can be graded into various levels of albuminuria. For intestinal schistosomiasis, although blood can be seen directly in the stool in frank cases, faecal occult blood (FOB) tests can be used to detect its presence with greater sensitivity.
National control programmes (NCPs): disease intervention programmes operating at a national level where a formal commitment in terms of policy, finances, staffing, and timetabling of a country to adopt and scale-up PC to a country-wide application. Measurement and assessment of progress towards reductions in selected disease targets are an obligatory requirement of the monitoring and evaluation component.
Neglected tropical diseases (NTDs): a group of several tropical diseases that collectively cause significant mortality and morbidity, often in impoverished rural communities. Schistosomiasis, alongside other helminthiases, is a key NTD and can be controlled by PC.
Praziquantel (PZQ): an oral anthelminthic originally discovered by Merck (Germany) during the 1970s whilst screening for tranquilisers with later codevelopment with Bayer (Germany). Initially intended for veterinary use, this safe broad-spectrum dewormer became the treatment of choice for schistosomiasis, particularly after off-patent generic drug production reduced purchasing costs to less than US$0.05 per 600 mg tablet. The drug is formally licensed and labelled for medical use for adults and children (aged 4 and above in years), at 40 or 60 mg/kg dosing for schistosomiasis, although lower dosing is advised for other trematodiasis. There is, however, no formal evidence capturing pharmacokinetics and pharmacodynamics in children.
Preventive chemotherapy (PC): a community-based treatment strategy, as endorsed by the WHO, where single administration of deworming medications such as, ivermectin, albendazole, and praziquantel, are regularly (co)administered within communities at significant risk of NTDs often by non-medical personnel. The intention is that by providing treatment future morbidity is prevented and disease transmission significantly reduced. For schistosomiasis children of school age are typically the major focus of treatment targeting.
Schistosomiasis: a water-borne parasitic disease that can be caused by six species of schistosome. In Africa, two forms of the disease, urogenital and intestinal, are dominant being caused by *Schistosoma haematobium* and *Schistosoma mansoni*, respectively. As part of its intricate life cycle, the schistosome needs to develop in freshwater snails. The cercaria, a short-lived larval stage emerging from the snail, is able to penetrate human skin directly.
